# Introduction to the Special Issue: New Frontiers in Acrylamide Study in Foods—Formation, Analysis and Exposure Assessment

**DOI:** 10.3390/foods9101506

**Published:** 2020-10-21

**Authors:** Cristina Delgado-Andrade, Marta Mesías, Francisco J. Morales

**Affiliations:** Institute of Food Science, Technology and Nutrition (ICTAN), Spanish National Research Council (CSIC), E-28040 Madrid, Spain; cdelgado@ictan.csic.es (C.D.-A.); fjmorales@ictan.csic.es (F.J.M.)

**Keywords:** acrylamide, chemical process contaminants, Maillard reactions, food safety, risk/benefits, mitigations, exposure

## Abstract

Acrylamide is a chemical contaminant that naturally originates during the thermal processing of many foods. Since 2002, worldwide institutions with competencies in food safety have promoted activities aimed at updating knowledge for a revaluation of the risk assessment of this process contaminant. The European Food Safety Authority (EFSA) ruled in 2015 that the presence of acrylamide in foods increases the risk of developing cancer in any age group of the population. Commission Regulation (EU) 2017/2158 establishes recommended mitigation measures for the food industry and reference levels to reduce the presence of acrylamide in foods and, consequently, its harmful effects on the population. This Special Issue explores recent advances on acrylamide in foods, including a novel insight on its chemistry of formation and elimination, effective mitigation strategies, conventional and innovative monitoring techniques, risk/benefit approaches and exposure assessment, in order to enhance our understanding for this process contaminant and its dietary exposure.

Chemical process contaminants are substances formed when foods undergo chemical changes during processing, including heat treatment, fermentation, smoking, drying and refining. Although necessary for making food edible and digestible, heat treatment can have undesired consequences leading to the formation of heat-induced contaminants such as acrylamide. It is well-established that acrylamide is formed when foods containing free asparagine and reducing sugars are cooked at temperatures above 120 °C in low moisture conditions. It is mainly formed in baked or fried carbohydrate-rich foods, as the relevant raw materials contain its precursors. These include cereals, potatoes and coffee beans. In 1994, acrylamide was classified by the International Agency for Research on Cancer as being probably carcinogenic to humans (group 2A), and in 2015, the European Food Safety Authority (EFSA) confirmed that the presence of acrylamide in foods is a public health concern, requiring continued efforts to reduce its exposure.

This special edition assembled nine quality papers, one review and eight research papers, focusing on several acrylamide-related issues, from raw materials to consumer exposure.

Different approaches for acrylamide determination in foods have been critically reviewed by Pan et al. [1], including conventional instrumental analysis methods and the new rapid immunoassay and sensor detection procedures. Advantages and disadvantages of different analysis technologies are compared in order to provide new ideas for the development of more efficient and practical analysis methods and detection equipment. Fernandes et al. [2] set up a high-resolution orbitrap mass spectrometry method for acrylamide measurement, with good repeatability, limit of detection and quantification, as well as enhanced detection sensitivity.

Some of the papers included have focused on the importance of precursor levels in the raw matter and the processing conditions on acrylamide formation. In this sense, Sun and colleagues [3] investigated the effects of nitrogen rate and storage time on potato glucose concentrations in different cultivars, analyzing the relationships between acrylamide, glucose, and asparagine for new cultivars. Mesias et al. [4] evaluated browning, antioxidant capacity and the formation of acrylamide and other heat-induced compound at different stages during the production of block panela (non-centrifugal cane sugar), establishing the juice concentration step as the critical point to settle mitigation strategies. Lee and co-workers [5] assessed the effects of thawing and frying methods on the formation of acrylamide and polycyclic aromatic hydrocarbons (PAHs) in chicken meat. They conclude that air frying could reduce the formation of acrylamide and PAHs in this food matrix at in comparison with deep-fat frying. In the case of cereal-derived products, Fernandes et al. [2] compared the acrylamide levels of biscuits with several production parameters, such as time/cooking temperature, placement on the cooking conveyor belt, color, and moisture. They state that the composition of the raw materials is the most important factor in the acrylamide content; therefore, establishing the level of precursor of ingredients strongly would contribute to the establishment of effective mitigation strategies. Industrial strategies to reduce acrylamide formation in Californian-style green ripe olives were studied by Martín-Veltedor et al. [6], with interesting results for the table olive industry to identify critical points in the production of this type of olives, thus helping to control acrylamide formation in this foodstuff.

It is well-known that potato- and cereal-derived products as well as coffee are important acrylamide sources in the Western diet. The food industry is especially interested in prospective studies dealing with the presence of acrylamide in these elaborations and its evolution in recent years. The study by Mesias et al. [7] evaluated acrylamide levels in seventy potato crisp samples commercialized in Spain with the purpose of updating knowledge about the global situation in this snack sector and evaluate the effectiveness of mitigation strategies applied, especially since the publication of the 2017/2158 Regulation. Results demonstrated that average acrylamide content in 2019 was 55.3% lower compared to 2004, 10.3% lower compared to 2008 and very similar to results from 2014, evidencing the effectiveness of mitigation measures implemented by Spanish potato crisp manufacturers. However, 27% of samples exhibited concentrations above the benchmark level established in the Regulation, which suggests that efforts to reduce acrylamide formation in this sector must continue. The same research team also developed a survey in 730 Spanish households to identify culinary practices which might influence acrylamide formation during the domestic preparation of French fries and their compliance with the acrylamide mitigation strategies described in the same document [8]. They conclude that although habits of the Spanish population are in line with recommendations to mitigate acrylamide during French fry preparation, educational initiatives disseminated among consumers would reduce the formation of this contaminant and, consequently, exposure to it in a domestic setting. Finally, an assessment of healthy and harmful Maillard reaction products (melanoidins and acrylamide) in a sun-dried coffee cascara beverage was developed by Iriondo-DeHond et al. [9], analyzing its safety and health-promoting properties. The novel beverage is proposed as a potential sustainable alternative for instant coffee, with low caffeine and acrylamide levels and a healthy composition of nutrients and antioxidants.

We hope that this Special Issue will be interesting for researchers engaged in the acrylamide issue in foods, including a novel insight on its chemistry of formation and elimination, effective mitigation strategies, classical and novel monitoring techniques, risk/benefit approaches, and exposure assessment, in order to enhance our understanding for this process contaminant and its dietary exposure.
